# Epidermal Growth Factor (EGFR) copy number aberrations in esophageal and gastro-esophageal junctional carcinoma

**DOI:** 10.1186/s13039-015-0181-0

**Published:** 2015-10-17

**Authors:** Åsa Dahle-Smith, David Stevenson, Doreen Massie, Graeme I. Murray, Susan J. Dutton, Corran Roberts, David Ferry, Aileen Osborne, Caroline Clark, Russell D. Petty, Zosia Miedzybrodzka

**Affiliations:** Division of Applied Medicine, University of Aberdeen, Aberdeen, AB25 2ZD UK; Department of Medical Genetics, Aberdeen Royal Infirmary, Aberdeen, AB25 2ZD UK; Department of Pathology, University of Aberdeen, Aberdeen, AB25 2ZD UK; Centre for Statistics in Medicine (CSM), Nuffield Department of Orthopaedics, Rheumatology and Musculoskeletal Sciences, University of Oxford, Botnar Research Centre, Windmill Road, Oxford, OX3 7LD UK; Eli Lilly and Company, 440 Route 22 East, Bridgewater, NJ 08807 USA; Department of Medical Genetics, University of Aberdeen, Aberdeen, AB25 2ZD UK

**Keywords:** EGFR Copy Number, Esophageal Carcinoma, Esophago-gastric junctional carcinoma

## Abstract

**Background:**

Clinical trials of agents targeting epidermal growth factor receptor (EGFR) in esophageal carcinoma (EC) have indicated a minority subgroup responsive to anti-EGFR therapies. Other investigations suggest increases in EGFR copy number are associated with poor prognosis in EC, but have used a variety of different techniques and tested numbers remain small. A validated assay for EGFR copy number in EC is needed, to allow investigation of EGFR copy number gain as a predictive biomarker for the anti-EGFR responsive subgroup of patients. We developed a scoring system in EC based upon established systems for EGFR fluorescence in-situ hybridisation (FISH) in lung cancer, and applied this in a series of 160 UK patients with advanced EC.

**Results:**

Dual colour FISH on formalin fixed paraffin embedded (FFPE) biopsies were scored independently by two operators as: disomy (score = 1), low trisomy (score = 2), high trisomy (score = 3), low polysomy (score = 4), high polysomy (score = 5) and amplification (score = 6). EGFR FISH positive cases (scores 5 and 6) were found in 32/160 (20 %) tumours, with high polysomy in 22 (13.8 %) and amplification in 10 (6.3 %). Two independent operator scores for FISH positivity were 100 % concordant. EGFR FISH positive status was not associated with clinic-pathological features. EGFR amplification was associated with worse survival (HR = 2.64, 95 % CI 1.04 to 6.71, *p* = 0.03).

**Conclusion:**

Our FISH scoring system for EGFR in advanced EC identifies a significant subgroup (20.0 %) of FISH positive patients. EGFR amplification, which is found in 6.3 %, is associated with poor survival. It is not known if there is a role for EGFR targeted treatment in this subgroup of patients, however we are now utilising this EGFR FISH assay and scoring system in biopsies from clinical trials utilising anti-EGFR targeted therapies.

## Background

Esophageal carcinoma (EC) is the eighth most common malignancy worldwide [1]. The incidence of esophageal adenocarcinoma (EA), has rapidly increased in the USA, Europe and Australia over the last 30-50 years [2]. Esophageal squamous cell carcinoma (ESCC) remains common in Western countries and is the most frequent histological subtype in the developing world, and the Middle and Far East [1, 3–5]. Despite advances in imaging modalities, surgical technique, chemotherapy and radiotherapy, survival remains poor, with USA and European five year survival rates of 24.5-39.6 % in patients presenting with localised disease and 9.8-17.5 % in the overall EC population [6, 7]. Chemotherapy confers only modest benefit in metastatic disease with median survival of only 9-11 months [8], and only half of patients will complete first line treatment due to toxicity or disease progression, although 40 % of patients may be fit for second line treatment [9, 10].

There has been a recent paradigm shift in the treatment of esophagogastric cancer. In the TOGA study, Bang and colleagues demonstrated survival benefit using the HER-2 monoclonal antibody trastuzumab over placebo with capecitabine/5-FU and cisplatin chemotherapy in patients with advanced gastro-esophageal junction or gastric adenocarcinomas overexpressing HER-2 by immunohistochemistry or *HER-2* gene amplification by FISH [11].

The primary end point of overall survival was met with median OS of 13.8 months in the trastuzumab arm compared to 11.1 months in the chemotherapy alone arm, *p* = 0.046. Patients whose tumours had either very high HER-2 over expression (IHC3+) or *HER-2* amplification confirmed by FISH, achieved a median survival of 17.9 months when treated with trastuzumab plus chemotherapy, compared to median survival of 12.3 months when treated with chemotherapy alone, HR 0.57 (95 % CI 0.41-0.81) [11].

Following this proof of concept of a role for targeted therapies in esophagogastric cancer, the clinical utility of novel agents targeting other growth factors have been investigated in EC.

There are two main classes of targeted therapy; small molecule tyrosine kinase inhibitors which act intra-cellularly on tyrosine kinases to prevent induction of downstream signalling pathways; and monoclonal antibodies which either compete with extracellular ligands to bind onto growth factor receptors or act directly on ligands to prevent ligand binding.

Targeted therapies against the Epidermal Growth Factor Receptor (EGFR) are in routine clinical use. Activation of the EGFR pathway stimulates intracellular signalling cascades including the RAF-MEK-ERK pathway which is involved in regulation of cell cycle progression, cell differentiation, proliferation and apoptosis, the subject of detailed reviews by Neuzillet *et al.* and McCrubrey *et al.* [12,13]. Activation of the EGFR pathway also initiates the PI3K-PTEN-Akt pathway, which has key roles in regulation of apoptosis and protein synthesis [14,15]. The EGFR and its ligands represent ‘druggable’ targets which when inhibited result in downregulation of growth factor pathways and thus anti-cancer effect. The tyrosine kinase inhibitor gefitinib has demonstrated survival benefit in *EGFR* mutated non-small cell lung cancer [16] and cetuximab, an anti-EGFR monoclonal antibody, improves survival in *KRAS* wild type colorectal cancer [17].

Recent trials of the anti-EGFR monoclonal antibodies cetuximab and panitumumab in combination with chemotherapy have not shown any overall survival benefit in EC [18,19]. The COG study, a phase III, randomised, double blinded trial of gefitinib, a tyrosine kinase inhibitor targeting EGFR, versus placebo in the second line setting of esophageal cancer patients did not demonstrate improvement in overall survival. However, median progression free survival with gefitinib was significantly improved from 1.1 to 1.57 months (HR 0.8, 95 % CI 0.66 to 0.96, *p* = 0.02) as was disease control rate, 24.1 % compared to 15.6 % at eight weeks (*p* = 0.016) [20]. Patient reported outcomes were also significantly improved. This suggests an anti-EGFR therapy responsive subgroup and highlights the importance of developing a predictive biomarker for anti-EGFR treatment benefit [20].

Gene copy number changes are frequent in EC in comparison to other tumour types including tumours in the gastrointestinal tract, even in the stomach [21]. Previous studies of EGFR gene copy number changes have suggested an association with poor prognosis in EC [21]. We thus propose that *EGFR* gene copy number changes might prove useful as predictive biomarkers for targeted therapies against EGFR [22].

*EGFR* copy number gain, including amplification, in esophageal and gastric cancers has been identified using several different methods, which used differing levels of gain for reporting *EGFR* amplification and inconsistent results regarding whether this confers a poor prognosis (Table 1) [23–32]. Differing classification systems for significant copy number gain or amplification, distinct biological differences between gastric cancer and EC, and some studies not assessing correlation between *EGFR* copy number and survival may account for reported disparities. In addition, the majority of previous studies have been small and undertaken in patients of differing ethnicities, and several studies were performed with technology that is no longer in widespread clinical use (Table 1) [23–32]. No previous study has provided a classification for *EGFR* copy number in EC to the degree that has become routine practice in other tumour types.

**Table 1:** Frequency of EGFR gene amplification in esophago-gastric cancer

Histology	Ethnicity	Technique	Amplification classification	Amplification (%)	Amplification impact on prognosis	Reference
GC	European	Southern Blot	Not documented	2/30 (6.7 %)	Not assessed	Lemoine 1991 [23]
GC	Chinese	FISH	≥ 15 EGFR copies in ≥10 % tumour cells OR ≥40 % cells with ≥4 EGFR copies OR EGFR/CERP7 ratio ~ 1 but cluster of ≥4EGFR copies in ≥10 % cells OR EGFR/CERP7 ratio ≥2 and cluster of ≥4EGFR copies in ≥10 % cells	20/69 (29 %)	Not assessed	YK 2011 [24]
GC	European	FISH	EGFR/ CEP 7 ratio ≥ 2.0	4/82 (4.88 %)	Poorer survival of EGFR amplified cases in multivariate analysis (HR 4.82, 95 % CI 1.32-17.7, *p* = 0.0176)	Kandel 2014 [25]
ESCC	Japanese	Southern Blot	EGFR/ CEP 7 ratio ≥ 2.0	9/42 (21.4 %)	Not assessed	Itakura 1994 [26]
ESCC	Thai	FISH	Low level: ratio 1.3-2.0, High level: ratio >2.0	8/15 (49 %)	No significant difference in survival in EGFR amplified cases	Sunpaweravong 2005 [27]
ESCC	Japanese	FISH/CGH	FISH: EGFR/CEP 7 ratio ≥ 2.0; CGH: >4 copies of EGFR gene	16/244 (7 %)	No significant difference in survival in EGFR amplified cases	Kato 2013 [28]
ESCC	Japanese	FISH	Low level: 3-10 EGFR signals/cell; High level: cluster of EGFR signals/>10 signals per cell	15/83 (18.1 %)	No significant difference in survival in EGFR amplified cases	Hanawa 2006 [29]
EA and ESCC	European	CISH	CISH + ve: >50 % cells with either tight EGFR clusters or > 4 EGFR copies per cell	2/16 (12.5 %)	Not assessed	Janmaat 2006 [30]
EA	European	FISH	Ratio ≥ 2.0 or presence of tight EGFR gene clusters	7/112 (6.25 %)	Poorer survival of EGFR amplified cases in multivariate analysis (*p* = 0.0004)	Marx 2010 [31]
EA	N. American NOS	Southern Blot	Ratio ≥ 2.0	7/87 (8.0 %)	Not assessed	Miller 2003 [32]

*Abbreviations*: *CGH* comparative genomic hybridisation, *CISH* chromogentic in situ hybridisation, *FISH* fluorescence in-situ hybridisation, *GC* gastric cancer, *EA* esophageal adenocarcinoma, *ESCC* esophageal squamous cell carcinoma

FISH is widely recognised as the “gold standard” diagnostic method for assessing gene copy number gain in human cancers. FISH for EGFR copy number alterations has been most extensively studied in formalin fixed paraffin embedded (FFPE) non-small cell lung cancer samples. Hirsch and colleagues [33] described four distinct categories in an analysis of 183 cases: disomy, trisomy, polysomy and amplification. Amplification was further classified as low (EGFR/ CEP7 ratio 2.1-3.0) or high (ratio >3.0). Significant correlation between EGFR gene copy number by FISH and EGFR protein expression by immunohistochemistry was identified (*p* < 0.001); high gene copy number also showed a trend towards poorer prognosis.

This principle of gene copy number classification in lung cancer was developed further by Cappuzzo *et al*, categorising *EGFR* FISH status into six categories with precise inclusion criteria of FISH positive cases comprising either high polysomy (≥40 % of cells with ≥ 4 copies of the *EGFR* gene) or amplification [34]. The concept of intratumoural heterogeneous amplification was also introduced, with the amplification criteria expanded from having an overall ratio of >2.0 to include either clustered *EGFR* signal with ratio of ≥2.0 or ≥15 *EGFR* copies in ≥10 % cells analysed. In a multivariate analysis using this criteria, *EGFR* FISH positive tumour status demonstrated significantly improved response rates (36 % in *EGFR* FISH positive versus 3 % in *EGFR* FISH negative patients; *p* < 0.001) and overall survival (median overall survival 18.7 months in EGFR FISH positive versus 7.0 months in *EGFR* FISH negative patients; *p* = 0.003) in 103 patients with non-small cell lung cancer being treated with the EGFR tyrosine kinase inhibitor gefitinib.

Varella-Garcia extended the *EGFR* FISH positive criteria to include larger and brighter *EGFR* signals compared to CEP7 signal in >10 % of tumour tissue with normal size *EGFR* signal in adjacent non- malignant cells, and recommended that fifty cells should be analysed in four distinct tumour areas [35]. An update providing guidance regarding sample storage and preparation was issued in 2009 [36].

In light of the potential importance of EGFR as a target in EC and *EGFR* copy number as a predictive biomarker, we adapted the consensus *EGFR* copy number analysis FISH assay used in non-small cell lung cancer as an assay for use in clinical trials and diagnostics in EC. Here we present the findings from applying this assay to FFPE tissue from 160 patients with advanced EC, approximately half of whom received second line treatment.

## Results

### Patient characteristics

Tumour samples were collected from 160 UK patients with pre-treated advanced esophageal or type I-II esophago-gastric junctional tumours. The majority of samples were diagnostic biopsies from the primary tumour (88.8 %), with surgical resection specimens accounting for 10.6 % and one case (0.6 %) was from a diagnostic liver biopsy. Clinico-pathological features are summarised in Table 3.

### EGFR FISH analysis

All FISH positive cases (*EGFR* amplified and high polysomy) scored by two independent observers were concordant (100 %). Sub-classification of FISH negative scores was also highly concordant, with a third scorer needed to agree classification in only 15/160 cases (9.4 %).

**Fig. 1:**
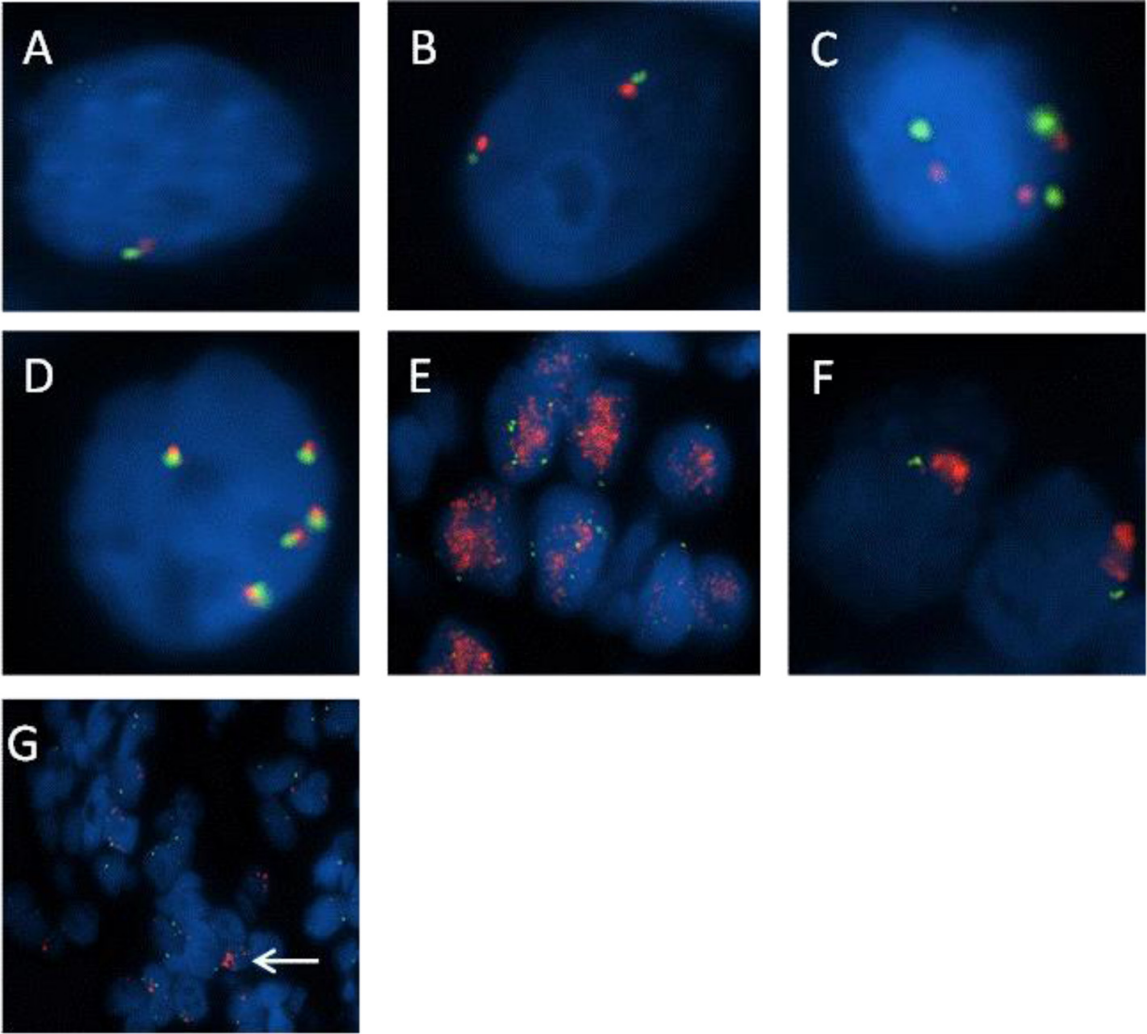
EGFR FISH classifications and patterns of EGFR FISH amplification observed. Using dual colour FISH and fluorescent microscopy, *EGFR* gene copy number was determined in 160 patients with advanced oesophageal cancer. *EGFR* FISH negative cases are present in panels **a**-**c**, *EGFR* FISH positive cases in panels D-F and a heterogeneously amplified case in panel G. The *EGFR* FISH classification as follows: loss of chromosome 7 (**a**), disomy (**b**), trisomy (**c**), high polysomy (**d**), amplification (double minute type) (**e**), amplification (homogenous staining region type) (**f**) heterogeneous amplification (**g**). Blue = DAPI nuclear staining; red signal = *EGFR*; green signal = chromosome 7 centromere; white arrow = EGFR amplified cells in a heterogeneous amplified case

Based on the classification criteria described in Table 2, 128/160 (80.0 %) of cases were classified as FISH negative and 32/160 (20.0 %) were classified FISH positive. In the *EGFR* FISH negative group, disomy was present in 45 cases (28.1 %); low trisomy in 47 (29.4 %); high trisomy in two cases (1.3 %) and low polysomy in 34 cases (21.3 %). Of the *EGFR* FISH positive cases, high polysomy was in displayed in 22 (13.8 %) cases and 10 (6.3 %) cases harboured *EGFR* amplification (Fig. 1).

**Table 2:** EGFR FISH classification system adapted from Varella-Garcia [35]

Tumour classification	EGFR status
Disomy: ≤2 copies in ≥90 % cells	Negative
Low trisomy: ≤2 copies in ≥40 % cells, 3 copies in 10-40 % cells, : ≤4 copies in <10 % cells	Negative
High trisomy: ≤2 copies in ≥40 % cells, 3 copies in ≥40 % cells, : ≤4 copies in <10 % cells	Negative
Low polysomy: ≥4copies in 10-40 % cells	Negative
High polysomy: ≥4copies in ≥40 % cells	Positive
Amplification: Any of	Positive
-EGFR/CEP7 ratio ≥2	
- Small cluster (4-10 copies) or innumerable tight clusters in >10 % cells	
- Larger and brighter EGFR signals v CEP7 signals in >10 % cells, with EGFR smaller than CEP7 in adjacent non-tumour cells	
- >15 copies of EGFR signal in >10 % tumour cells INDEPENDANT of EGFR/CEP7 ratio	
If amplified, pattern of amplification:	
- Homogenous staining region (HSR)	
- Double minute (DM)	
- Heterogeneous amplification (HA)

Three patterns of amplification were observed, double minutes, homogenous staining region and heterogeneous amplification (Fig. 1). Double minutes and homogenous staining regions in interphase have previously been described by Martin and colleagues [37]. Most amplified cases displayed uniform amplification with either a diffuse specked signal pattern consistent with double minutes or tightly packed signal clustering representing a homogenous staining region. A homogenous staining region is caused by amplicon clustering on a chromosome whereas double minute amplification is due to multiple copies of non-centromeric chromosomal fragments containing EGFR, seen as disseminated signals. Intra-tumoural heterogeneity for amplification was also observed, and tumours with ≤50 % of cells exhibiting amplification were classed as heterogeneous amplification. Of the ten amplified cases, five were homogenous staining regions, three cases heterogeneously amplified and two were double minutes.

### Association of *EGFR* FISH status with clinical and pathological variables

Using Fisher’s exact test, no association was demonstrated between *EGFR* FISH status and gender (*p* = 0.752), disease site (*p* = 0.422), performance status (*p* = 0.085), body mass index (*p* = 0.737), brain metastases (*p* = 0.361) or number of prior chemotherapy regimens (*p* = 0.406) (Table 3). No association was detected between central review of histology (one patient with baseline undifferentiated carcinoma was excluded from analysis) using Pearson X^2^ test (*p* = 0.909).

**Table 3:** Association of EGFR FISH status with clinicopathological features

	All patients (*N* = 160)	EGFR FISH negative (*N* = 128)	EGFR FISH positive (*N* = 32)	P value
Age (mean,SD)	64.02 (9.49)	64.15 (9.64)	63.49 (8.99)	0.725
Gender:				
Male	133 (83.1 %)	10.7 (80.5 %)	26 (19.5 %)	0.752
Female	27 (16.9 %)	21 (77.8 %)	6 (22.2 %)	
Histology:				
Adenocarcinoma	118 (73.8 %)	94 (79.7 %)	24 (20.3 %)	0.909
Squamous cell carcinoma	41 (25.6 %)	33 (80.5 %)	8 (19.5 %)	
Undifferentiated carcinoma	1 (0.6 %)	1 (0.6 %)	0 (0.0 %)	
Disease site:				
Oesophagus	124 (77.5 %)	100 (80.6 %)	24 (19.4 %)	0.422
Type I junctional	16 (10.0 %)	14 (87.5 %)	2 (12.5 %)	
Type II junctional	20 (12.5 %)	14 (70.0 %)	6 (30.0 %)	
WHO PS:				
0 (Asymptomatic)	35 (21.9 %)	32 (91.4 %)	3 (8.6 %)	0.085
1 (Symptomatic but ambulatory)	91 (56.9 %)	72 (79.1 %)	19 (20.9 %)	
2 (Symptomatic but resting <50 % of day)	34 (21.3 %)	24 (70.6 %)	10 (29.4 %)	
Body Mass Index category:				
Underweight	15 (9.4 %)	13 (86.3 %)	2 (13.3 %)	0.737
Normal	83 (51.9 %)	66 (76.5 %)	17 (20.5 %)	
Overweight	37 (23.1 %)	28 (75.7 %)	9 (24.3 %)	
Obese	17 (10.6 %)	15 (88.2 %)	2 (11.5 %)	
Missing data	8 (5.0 %)			
Brain metastases				
No	158 (98.8 %)	127 (80.4 %)	31 (19.6 %)	0.361
Yes	2 (1.3 %)	1 (50.0 %)	1 (50.0 %)	
No. of previous chemotherapies:				
1	103 (64.4 %)	83 (80.6 %)	20 (19.4 %)	0.406
2	50 (31.1 %)	38 (76.0 %)	12 (24.0 %)	
3	7 (4.4 %)	7 (100.0 %)	0 (0.0 %)	

*Abbreviations*: *WHO PS* World Health Organisation Performance Status

### Association between *EGFR* FISH status and survival

**Fig. 2:**
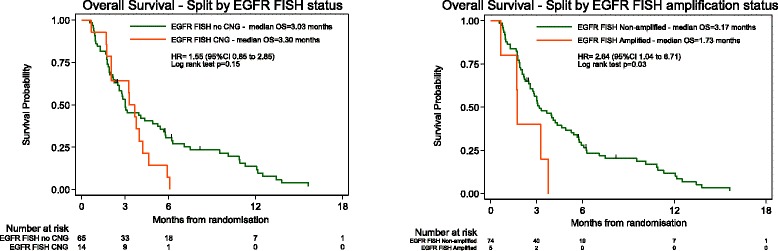
Kaplan-Meier survival analysis of patients with advanced oesophageal cancer stratified according to EGFR FISH classification performed in 79 patients with advanced oesophageal cancer who were receiving best supportive care. There was a non-significant trend towards poorer overall survival (OS) in patients with EGFR copy number gain (≥4 EGFR copies in ≥40 % cells) compared to those with no EGFR copy number gain; HR 1.55, 95 % CI 0.85 to 2.85, median OS 3.30 v 3.03 months *p* = 0.15. Kaplan-Meier survival analysis in this patient group demonstrated poorer OS in patients with EGFR amplification compared to EGFR non-amplified cases; HR 2.64, 95 % CI 1.04 to 6.71, median OS 1.76 v 3.17 months, *p* = 0.03

In order to remove the effects of treatment interaction, the relationship between *EGFR* gene copy number and overall survival in the 79 of 160 patients that received no further treatment was examined. This allowed the therapy independent prognostic impact of *EGFR* FISH status to be examined in the population of advanced oesophageal cancers that have been, and most likely will be, evaluated in future clinical trials of anti-EGFR agents. There was no significant difference in overall survival in *EGFR* FISH positive (*N* = 14) versus negative (*N* = 65) patients, (HR 1.55, 95 % CI 0.85 to 2.85, median overall survival 3.30 v 3.03 months *p* = 0.15, Fig. 2), but there is limited power to detect anything except a large difference due to the small numbers. EGFR amplified cases (*N* = 5) had significantly worse overall survival compared to EGFR non-amplified cases (*N* = 74), (HR 2.64, 95 % CI 1.04 to 6.71, median overall survival 1.76 v 3.17 months, *p* = 0.03, Fig. 2).

There was no significant difference in overall survival between *EGFR* FISH positive and *EGFR* FISH negative cases, or between *EGFR* amplified and non-amplified cases in the whole cohort and in patients that received a variety of further treatments (data not shown).

## Discussion

In this study we successfully applied an *EGFR* FISH classification previously developed for non-small cell lung cancer, for use in esophageal cancer and characterised a series of 160 FFPE samples using dual colour probe *EGFR* FISH. Scoring in all FISH positive cases (amplified and high polysomy; 5 and 6) was concordant between two independent observers, and very high (90.6 %) for individual FISH negative categories 1 to 4, with concordance reached in all cases following third independent scorer analysis. Survival analysis in patients receiving best supportive care using this classification demonstrated worse prognosis in FISH amplified cases (*p* = 0.03).

In our series, 20 % of EC patients were EGFR FISH positive, representing a significant subgroup of patients with advanced esophageal cancer with potential upregulation of and growth dependency upon the EGFR pathway. The frequency of *EGFR* amplification present in 6.3 % (ten cases) is consistent with previous reports (6.25-49 %) [23–32]. The frequency of FISH amplification is similar to that seen for the HER-2 receptor [28,38].

Three distinct patterns of amplification were observed, homogeneous staining regions representing large, bright signals caused by amplicon clustering; double minutes demonstrating fragments of non-centromeric chromosome material [37]; and heterogeneous amplification cases of both double minutes and homogeneous staining region type. Cases were felt to be heterogeneously amplified if ≤50 % tumour cells demonstrated amplification. The issue of intratumoural heterogeneity remains controversial. Different cut-off values of ≤50 % in gastric and 5-50 % in breast cancers to define heterogeneous HER-2 amplification [39,40] have been applied and although EGFR copy number heterogeneity has been identified in non-small cell lung cancer [41] and colorectal cancer [42] no standardised classification system has been developed. Cases meeting our *EGFR* amplification criteria have significantly worse overall survival compared with non-amplified cases (median overall survival 1.76 v 3.17 months, *p* = 0.03), in patients not treated with anti-cancer therapies, confirming EGFR amplification as a therapy independent prognostic biomarker in EC and supporting the use of dual colour EGFR FISH as a robust method of analysis of EGFR gene copy number. Further studies are required to determine whether *EGFR* amplification in EC is useful as a predictive biomarker to identify patients suitable for anti-EGFR targeted therapy and we suggest that the method and scoring system described here is fit for this purpose.

A lack of standardised biopsies is a limitation of our study, the majority of tumour samples were from diagnostic biopsies of the primary tumour (88.8 %), surgical resection specimens accounted for 10.6 % and one case (0.6 %) had a diagnostic biopsy from a metastatic hepatic deposit. Due to the invasive nature of endoscopy and biopsy, repeat tumour biopsies are rarely performed in this tumour type outside clinical trials or when patients are scheduled for surgery. Disease progression causing dysphagia requiring stent insertion is a potential opportunity for repeat biopsy, to differentiate, for example, stricture secondary to radiation induced fibrosis from tumour. Repeat endoscopies are not without risk and are considered intrusive for many patients and clinicians, particularly if it will not alter treatment options.

Previous studies of *EGFR* copy number in EC are summarised in Table 1. A variety of techniques have been employed to investigate the frequency of EGFR copy number gain or amplification in esophago-gastric cancer, yielding a frequency of 6.25- 49 % (Table 1) [16–25]. Of the studies using FISH to evaluate *EGFR* copy number change or amplification, two were in gastric cancer patients, demonstrating poor survival in the 4.88 % of European patients with amplification, however survival outcome was not assessed in 29 % EGFR amplified Chinese gastric cancer patients [24, 25]. In studies of EC where the prognostic impact of EGFR amplification by FISH has been assessed, there are discordant results, perhaps due to differing scoring criteria and histological tumour subtype [27–29, 31].

The distinct biological and molecular features of gastric cancer and EC, in particular the different frequencies of gene copy number changes, as well as the lack of a validated classification system for significant *EGFR* copy number gain or amplification, may account for inconsistencies in results. In addition, the majority of previous studies have been small and undertaken in patients of differing ethnicities and several studies were performed with technology that is no longer in widespread clinical use, making it unclear whether *EGFR* amplification does result in poorer survival.

The frequency of *EGFR* amplification may be lower in European and North American populations (6.5 -12.5 %) when compared to studies in Asian patients (7-49 %), implying a significant ethnic component to EGFR dysregulation in esophago-gastric cancer. Ethnic differences in molecular abnormalities have been identified in NSCLC where patients of Asian origin are more likely than Caucasians (35 v 11 %) to harbour the *EGFR* mutations inferring increased benefit from anti-EGFR therapy [16, 43, 44].

The limited benefit and high toxicity of multi-agent cytotoxic chemotherapies in esophago-gastric cancer have prompted investigation of targeted therapies, including those targeting EGFR and HER-2. Trastuzumab, a monoclonal antibody against HER-2, has demonstrated activity in combination with platinum doublet chemotherapy in the 10-15 % patients with gastric cancer and tumour HER-2 protein overexpression or amplification, compared to chemotherapy alone [11].

The COG study of gefitinib, versus placebo in the second line setting of esophageal cancer patients did not demonstrate improvement in overall survival in unselected patients. Median progression free survival with gefitinib was significantly improved from 1.1 to 1.57 months (HR 0.8, 95 % CI 0.66 to 0.96, *p* = 0.02) as was disease control rate, 24.1 % compared to 15.6 % at eight weeks (*p* = 0.016) [20]. The COG study results suggest that anti–EGFR tyrosine kinase inhibitor therapies represent a plausible therapeutic option for a sub-group of responsive patients. Accordingly, we propose that our scoring system should be used to explore the value of EGFR FISH positivity as a predictive biomarker for response to anti- tyrosine kinase inhibitor therapies, especially gefitinib [20].

## Conclusion

In conclusion, we have shown that *EGFR* amplification assessed using our standardised FISH scoring system is a therapy independent prognostic biomarker of poor outcome in EC and represents a practical, robust assay useful for clinical research and clinical practice, particularly for investigation as a predictive biomarker for anti-EGFR therapies in clinical trials.

## Methods

### Patient samples for FISH

Ethical approval was obtained from the North of Scotland Research Ethics Committee. Esophageal tumour blocks from 160 patients with advanced EC who had received prior chemotherapy treatment were analysed (AJCC 7^th^ Edition, stage IIIB/C or IV), of which 81 went on to receive further systemic therapy [45]. Survival was measured in months from day of diagnosis until death. No analysis of EGFR expression was performed due to insufficient tumour tissue being available. Karyotype analysis was not possible as this was a retrospective study using archival formalin fixed paraffin embedded tissue.

### Fluorescence in situ hybridisation (FISH)

Sections of 4 μm thickness cut from FFPE esophageal tumour blocks were mounted on positively charged slides and pre-treated according to manufacturer instructions (Vysis Paraffin Pre-Treatment Reagent Kit II, Abbott laboratories, Maidenhead, UK). Briefly, slides were baked overnight at 50 °C, then deparaffinised in xylene, followed by ethanol rehydration. They were then washed in Pre-Treatment solution for 10 min and de-ionised water for three minutes. Following this, slides were immersed in Protease Buffer II and Protease I solution (pepsin activity 1:3000-1:3500) for 25 min and after washing in de-ionised water, were dehydrated in an ethanol series. Once dry, 10 μl of Vysis EGFR/CEP7 dual colour probe (Abbott Laboratories) was applied and a coverslip fixed using rubber cement. The slides were then transferred to a ThermoBrite StatSpin® (Abbott laboratories) programmed to denature at 80 °C for six minutes, followed by hybridisation at 37 °C for 16 h.

Post- hybridisation, slides were washed in 2XSSC/0.3 % Igepal (Sigma-Aldrich Company Ltd Dorset, UK) and air dried in darkness. Once dried, nuclear counter stain containing 4’,6-diamidino-2-phenylindole (DAPI) (Vectashield mounting medium, Vector Laboratories, Peterborough, UK) was applied, and a new cover slip was attached. Slides were stored in darkness at 4 °C.

### FISH scoring

Analysis was performed by two independent scorers, using a fluorescent microscope (AXIO Imager M1, Carl Zeiss Microscopy, Cambridge, UK) and images recorded using CytoVision 7.3.1 software (Leica Biosystems, Newcastle, UK). Three areas of tumour were examined, with 20 cells counted in each area. EGFR FISH scores were assigned for FISH negative: disomy (score = 1), low trisomy (score = 2), high trisomy (score = 3) and low polysomy (score = 4) and for FISH positive: high polysomy (score = 5) and amplification (score =6) (Table 2). In cases of discordance between the first two scorers, further analysis was carried out by a third independent scorer and agreement reached.

### Statistical analysis

Relationships between baseline clinico-pathological features and EGFR mutation status were analysed using Pearson Chi^2^ or Fisher’s exact test when cell counts were ≤5. Hazard ratios with 95 % confidence intervals, log rank test and Kaplan–Meier curves were constructed comparing overall survival, defined as time from diagnosis to death) and progression free survival (defined as time from diagnosis to progression or death) in EGFR FISH positive and negative cases, and EGFR amplified cases versus all other cases in the 79 patients who did not receive further systemic treatment for their disease. Statistical analysis was performed using Stata Version 13.1 (StataCorp LP, Texas, USA).

## Abbreviations

EA: Esophageal adenocarcinoma
EC: Esophageal cancer
EGFR: Epidermal growth factor receptor
ESCC: Esophageal squamous cell carcinoma
FFPE: Formalin foxed paraffin embedded
FISH: Fluorescence in-situ hybridisation
HER-2: Human epidermal growth factor receptor 2
